# Recent Advancements in Liquid Metal Flexible Printed Electronics: Properties, Technologies, and Applications

**DOI:** 10.3390/mi7120206

**Published:** 2016-11-30

**Authors:** Xuelin Wang, Jing Liu

**Affiliations:** 1 Department of Biomedical Engineering, School of Medicine, Tsinghua University, Beijing 100084, China; wang-xl15@mails.tsinghua.edu.cn; 2 Beijing Key Lab of CryoBiomedical Engineering and Key Lab of Cryogenics, Technical Institute of Physics and Chemistry, Chinese Academy of Sciences, Beijing 100190, China

**Keywords:** liquid metal, flexible printed electronics, 3D printing, functional device, additive manufacture, consumer electronics

## Abstract

This article presents an overview on typical properties, technologies, and applications of liquid metal based flexible printed electronics. The core manufacturing material—room-temperature liquid metal, currently mainly represented by gallium and its alloys with the properties of excellent resistivity, enormous bendability, low adhesion, and large surface tension, was focused on in particular. In addition, a series of recently developed printing technologies spanning from personal electronic circuit printing (direct painting or writing, mechanical system printing, mask layer based printing, high-resolution nanoimprinting, etc.) to 3D room temperature liquid metal printing is comprehensively reviewed. Applications of these planar or three-dimensional printing technologies and the related liquid metal alloy inks in making flexible electronics, such as electronical components, health care sensors, and other functional devices were discussed. The significantly different adhesions of liquid metal inks on various substrates under different oxidation degrees, weakness of circuits, difficulty of fabricating high-accuracy devices, and low rate of good product—all of which are challenges faced by current liquid metal flexible printed electronics—are discussed. Prospects for liquid metal flexible printed electronics to develop ending user electronics and more extensive applications in the future are given.

## 1. Introduction

Printed electronics is the technology to fabricate electronic devices based on the principle of printing [1]. Unlike traditional printing technology, the inks used in the printing machine are electronic materials with the properties of being conductive, dielectric, semi-conductive, or magnetic. Printed electronics is closely related to diverse fields such as organic electronics, plastic electronics, flexible electronics, and paper electronics [2], which indicates that one can not only print circuits on silicon and glass, but also on plastic, paper, and more flexible substrates. One of the methods to produce flexible electronics is to directly print conductive materials onto flexible substrates.

Classical flexible electronics refers to the technology by which organic/inorganic electronic devices are deposited on a flexible substrate to form a circuit [3]. Although the rigid circuit board can protect the electronic components from being damaged, it restricts the ductility and flexibility of the electronics. With intriguing properties such as softness, ductility, and low-cost fabrication, flexible electronics has broad application prospects in the information, energy, medicine, and defense technology fields through providing smart sensors, actuators, flexible displays, organic light-emitting diodes (OLEDs), and so on. The most obvious characteristic of flexible electronics lies in their flexibility compared with traditional rigid microelectronics, which makes them stretchable, conformal, portable, wearable, and easy to print quickly [4,5,6,7,8]. Because of their unique merits in terms of electrical, printable, biomedical, and sensing properties, flexible electronics can find diverse applications in electronic components [9], printing technology, implantable devices [10], and health monitors [11], with specific uses such as antennas [12], eyeball cameras [13] and pressure sensors [14], etc. Nowadays, two common strategies have been adopted to improve the softness of the electronics [15]. One is embedding conductive materials, which are stiff and rigid, onto a stretchable substrate, such as poly(dimethylsiloxane) (PDMS). Rogers and coworkers introduced a complex wavy structure to keep the circuit stretchable, which can absorb the major tension when stress acts on the soft substrate [16,17]. The other is using inherently stretchable conductors to form the circuit [18]. Russo and coworkers demonstrated a way to connect the circuit using conductive silver ink, which can directly write conductive text to interconnect light-emitting diode (LED) arrays and three-dimensional (3D) antennas on paper [12].

Clearly, flexible printed electronics (FPE) combines features of flexible electronics and printed electronics. In this way, one can quickly manufacture functional flexible electronic devices. Along with the maturation of printing technology, flexible printing has become a hot topic in research. Several typical printing technologies have been emerging, such as tapping mode printing [18], aerosol jet printing, roll-to-roll technology [19], inkjet printing [20], and micropen and brush printing [21,22]. Among them, micropen is perhaps the simplest method: pump the electrical ink into a cartridge to directly write out conductive texts. This makes it possible to draw a circuit diagram on an A4 sheet of paper. A micropen evidently reduces the complexity of circuit production and improves the efficiency of electronic circuit manufacturing. FPE have contributed to significant achievements in different fields, such as flexible display devices [23], thin-film solar cells [24,25], large area sensors and drivers [26,27], electronic skin [28], wearable electronics and biological prosthetic devices [29], self-charging system [30], self-powered wireless monitoring [31], etc. If a circuit benefits from the properties of softness, conformability, stretchability and portability, one can call it a flectional electronic device. Meanwhile, many laboratories have developed electronic devices with flexibility, such as warped display [32], folding battery [9], soft field effect transistor [33], stretchable wire [34], and so on. FPE can also be used in health monitoring, medical examination, vital signs detection, and other daily life needs. Flexible biomedical equipment has been widely utilized in the field of implantable devices [10], nerve connection [35,36], health monitoring [11], and wearable medical devices. FPE has also been used for biological sensing, the most important application of which is electronic skin (E-skin), such as pressure mapping [37], self-healing sensor [38], prosthesis [39], pressure sensor [14], etc. There are many research organizations working on different materials applied on flexible electronic skin. In Bao’s laboratory, they invented a skin-inspired artificial mechanoreceptor system and the receptor can transform the pressure stimulation signal into electrophysiological signal that human neurons can perceive [40]. In Rogers’s laboratory, they initiated a wavy structure to realize stretchable epidermal electronics that can maintain the original shape and function after compressing or stretching [16,41,42], In Chiolerio’s laboratory, the researchers invented a printable spin-coated silver nanocomposite ink to manufacture a resistive switching devices for neuromorphic applications and a nanocomposite flexible liquid state device in a synthetic colloidal suspension [43,44].

**Figure 1:**
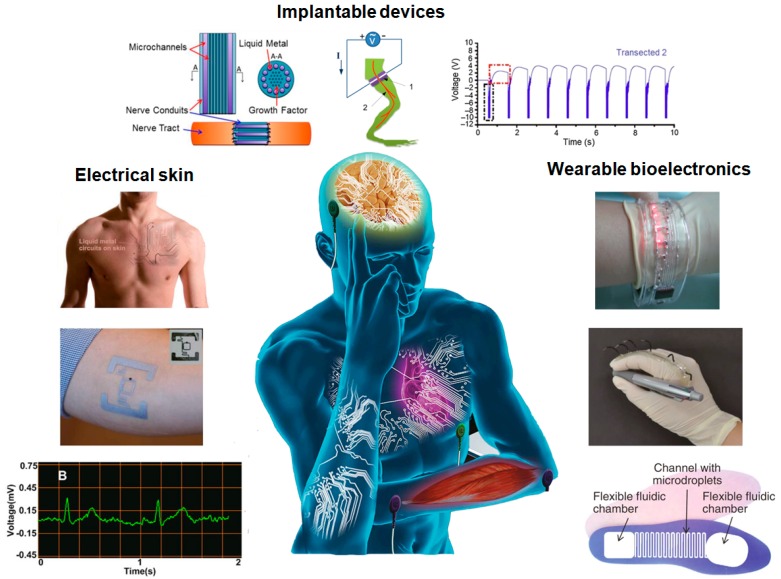
Personalized flexible printed electronics based on liquid metal. “Implantable devices”: Liquid metal nerve connection [35] (https://arxiv.org/abs/1404.5931); Injectable 3D bioelectrode [54] (Reproduced with permission from Jin, C.; Liu, J. et al., Injectable 3-D fabrication of medical electronics at the target biological tissues; published by Scientific Reports, 2013.); Electrophysiological measurement of liquid metal reconnected nerve [36]; “Electrical skin”: Liquid metal circuits on human body [55] (Reproduced with permission from Guo, C.; Liu, J. et al., Rapidly patterning conductive components on skin substrates as physiological testing devices via liquid metal spraying and pre-designed mask; published by Journal of Mterials Chemistry B, 2014.); Liquid metal tag on human skin [56] (Reproduced with permission from Jeong, S.H.; Wu, Z. et al., Liquid alloy printing of microfluidic stretchable electronics; published by Lab on a Chip, 2012.); ECG test by liquid metal electrode [57] (Reproduced with permission from Yu, Y.; Zhang, J.; Liu, J., Biomedical implementation of liquid metal ink as drawable ECG electrode and skin circuit; published by PLoS ONE, 2013.); “Wearable bioelectronics”: Liquid metal wristband [58] (Reproduced with permission from Wang, Q.; Yu, Y.; Yang, J.; Liu, J., Fast fabrication of flexible functional circuits based on liquid metal dual-trans printing; published by Advanced Material, 2015.); A wearable data glove [59] (Reproduced with permission from Matsuzaki, R.; Tabayashi, K., Highly stretchable, global, and distributed local strain sensing line using gainsn electrodes for wearable electronics; published by Advanced Functional Material, 2015.); Footwear-embedded microfluidic energy harvest [60] (Reproduced with permission from Krupenkin, T.; Taylor, J.A., Reverse electrowetting as a new approach to high-power energy harvesting; published by Nature Communication, 2011.).

Generally, traditional nanoparticle-based conductive ink does not have intrinsic conductivity, and needs special post-processing (e.g., sintering, annealing) to remove solvent from the conductive ink to achieve conductive capacity, such as silver nanoparticle, PEDOT:PSS, polyaniline-based ink, nickel and copper conductive ink [45,46,47,48,49]. The newly emerging liquid metal ink has intrinsically high conductivity, which enables it to be a kind of ideal conductive ink (Table 1). (In this paper, EGaIn refers to Ga–In, which contains the metal elements gallium and indium, commonly in a mixture of 75.5% gallium and 24.5% indium by weight; Galinstan refers to Ga–In–Sn, which contains the metal elements gallium, indium, and tin, commonly in a mixture of 62.5% gallium, 21.5% indium and 16% tin by weight.) Liquid metal, just as its name suggests, is a kind of metal that remains in liquid phase from room temperature up to 2000 °C. (However, 2000 °C is a generic number; it depends upon the composition.) It is superior to many other liquid materials in terms of thermal conductivity and resistivity at low temperatures or around room temperature [50]. One traditional liquid metal—mercury—is not accepted on account of its high toxicity to the human body. However, because of its non-toxicity and benign biocompatibility, a liquid metal eutectic alloy of gallium indium has made important progresses in the biomedical arena such as being used as the material of bone cement [51], as a vascular contrast agent [52], and in drug delivery nanomedicine [53]. All of these indicate that liquid metal can be widely used in the fields of electronics, materials, and biology, which significantly broadens its impact. Figure 1 shows the typical applications of personalized flexible printed electronics based on liquid metal, such as implantable devices [35,36,54], electrical skin [55,56,57] and wearable bioelectronics [58,59,60] etc. Along with the development of material science and technology, liquid metal flexible printed electronics are quickly shaping the field of flexible electronic circuits and allied machines, serving as a basic way of quickly making functional devices. In this review, we are dedicated to integrating the liquid metal alloy inks with flexible printed electronics and focus our attention on interpreting the recent advancements in liquid metal flexible printed electronics.

## 2. Basic Properties of Liquid Metal

Generally speaking, alloy elements of low melting point include gallium, bismuth, lead, tin, cadmium, and indium. Among them, GaIn_24.5_ is commonly used, which is a mixture of 75.5% gallium and 24.5% indium by weight. For the same composition, a different proportion may lead to quite varied properties in the alloys. Sometimes, even a small change of mass ratio would cause strong variation in the material behaviors. If loaded with certain microelements, the alloy may also display additional properties accordingly. Therefore, one can change the ratio of chemical materials of alloys or add some microelements to adjust the melting point and other properties to fulfill various specific needs. Room-temperature liquid metal material, generally represented by gallium and its alloys, offers a unique for to manufacturing flexible circuits, due to the combined nature of metallicity and fluidity [62,63], which makes it especially suitable for printing on soft substrates. (In this paper, the liquid metal alloy refers to gallium and its alloys.) From [62], one can get information on the main physical properties of frequently used liquid metal alloys, such as combination, melting point, density, resistivity, and thermal conductivity. In this review, we mainly discuss the resistivity, flexibility, adhesion, and wettability of liquid metal in flexible printed electronics.

### 2.1. Resistivity

Resistivity is the ability of an object to conduct an electric current, and generally the conductivity of metal is better than that of a non-metal. If the liquid metal is applied to print a flexible circuit, we can calculate the resistance value of a liquid metal wire by the formula:

(1) $$R=\frac{\rho \cdot L}{A},$$

where R is the resistance; ρ is the resistivity and for the same material ρ is a fixed value; *L* is the length of measured liquid metal conductive wire; and A is the cross-sectional area. As can be seen from Equation (1), the resistance of the material is directly proportional to the length of the material and the resistivity, and is inversely proportional to its cross-sectional area.

The relationship between the volume and length is:

(2) $$A=\frac{V}{L}.$$

Therefore, one can get the factors associated with resistance change:

(3) $$\frac{R}{R_{0}}=\frac{\frac{\rho \cdot L}{A}}{\frac{\rho_{0}\cdot L_{0}}{A_{0}}}=\frac{\rho V_{0}}{\rho_{0}V}{\left(\frac{L}{L_{0}}\right)}^{2}.$$

The volume of the liquid metal conductor is constant when drawing $V=V_{0}$; the resistivity is also considered constant, $\rho =\rho_{0}$.

Consequently, one can simplify Equation (3) to:

(4) $$\frac{R}{R_{0}}={\left(\frac{L}{L_{0}}\right)}^{2}.$$

The resistance variation of a liquid metal conductor has a linear relationship with the square of the length change of the conductor before and after stretching, confirming the relationship between resistance and tensile length, which provides theoretical support for the development of a liquid metal flexible sensor [55].

However, the resistivity c of the liquid metal has a significant relationship with the thermal conductivity [64,65]:

(5) $$\frac{\Lambda }{\rho T}=\frac{\pi^{2}\kappa_{B}^{2}}{3e^{2}}=2.45\times 10^{-8}(W\cdot {\Omega /K}^{2}).$$

**Figure 2:**
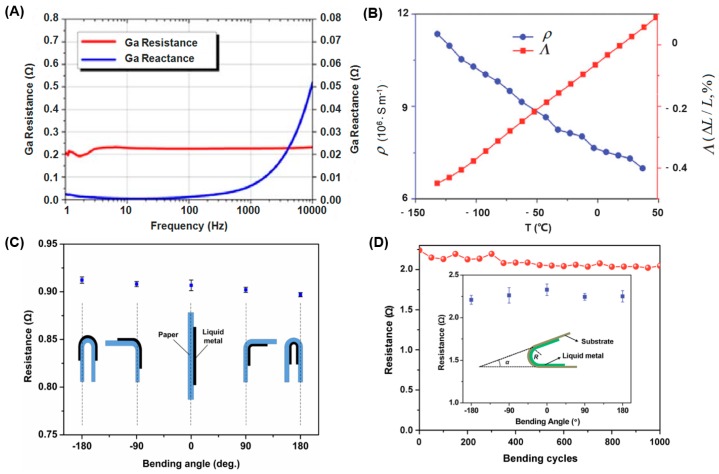
(**A**) The resistance and reactance curves from 1 Hz to 10 kHz of the liquid metal Ga [57] (Reproduced with permission from Yu, Y.; Zhang, J.; Liu, J., Biomedical implementation of liquid metal ink as drawable ECG electrode and skin circuit; published by PLoS ONE, 2013.); (**B**) ρ (the resistivity)-T (temperature) and Λ (the thermal conductivity)-T (temperature) curves of liquid metal Bi_35_In_48.6_Sn_16_Zn_0.4_ [64] (Reproduced with permission from Wang, L.; Liu, J., Compatible hybrid 3D printing of metal and nonmetal inks for direct manufacture of end functional devices; published by Science China Technological Sciences, 2014.); (**C**) resistance values of liquid metal line at five different bending angles [69] (Reproduced with permission from Zheng, Y.; He, Z.; Gao, Y.; Liu, J., Direct desktop printed-circuits-on-paper flexible electronics; published by Scientific Reports, 2013.); (**D**) resistance values of liquid metal line at different bending angles and different bending cycles [70] (Reproduced with permission from Wang, L.; Liu, J., Pressured liquid metal screen printing for rapid manufacture of high resolution electronic patterns; published by RSC Advances, 2015.).

Here, Λ is the thermal conductivity; ρ is the resistivity; and T is the thermodynamic temperature. Figure 2B shows the ρ−T and the Λ−T curves for Bi_35_In_48.6_Sn_16_Zn_0.4_. With the increase in temperature from −150 °C to 50 °C, Λ shows a linear increase while ρ shows a linear decrease. $\rho_{B}$ is the Boltzmann constant. e is the electron charge, $e=-1.6\times 10^{-19}C$. Equation (5) is the famous Wiedemann–Franz–Lorenz equation, from which one can deduce that the thermal conductivity of the liquid metal is proportional to the product of the resistivity and the thermodynamic temperature. One can get the resistance value of some number of wires connected in parallel by Ohm’s Law [66]:

(6) $$R={\left(\sum_{i=1}^{n}\frac{1}{R_{i}}\right)}^{-1}.$$

**Figure 3:**
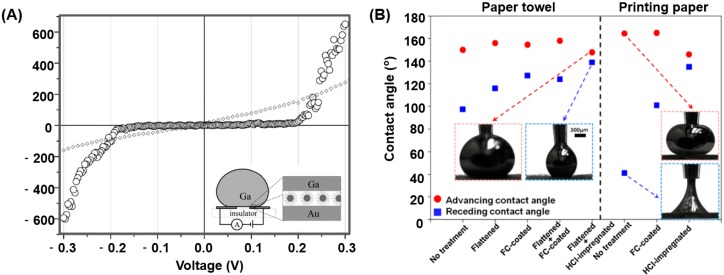
(**A**) Plot of the measured current and voltage of the electronic device composed of one gallium droplet and a layer of gold nanoparticles [68] (Reproduced with permission from Du, K.; Glogowski, E.; Tuominen, M.T.; Emrick, T.; Russell, T.P.; Dinsmore, A.D., Self-assembly of gold nanoparticles on gallium droplets: Controlling charge transport through microscopic devices; published by Langmuir, 2013.); (**B**) the advancing and receding contact angle of the liquid metal droplets on the paper towel and printing paper [71] (Reproduced with permission from Kim, D.; Lee, Y.; Lee, D.-W.; Choi, W.; Yoo, K.; Lee, J.-B., Hydrochloric acid-impregnated paper for gallium-based liquid metal microfluidics; published by Sensors and Actuators B: Chemical, 2015.).

Here, n is the number of wires connected in parallel. $R_{i}$ is the individual resistance values, i.e.,

(7) $$R_{i}=\rho \int_{0}^{L}\frac{dx}{A_{i}(x)},$$

where ρ is the resistivity and the resistivity of EGaIn is $29.4\;\times \;10^{-6}\;\Omega \cdot \mathrm{cm}$. Gozen et al. demonstrates that the measured and predicted resistance values show a high degree of agreement with the number of wires changing [66]. However, there are some non-Ohm’s Law phenomena that the current does not linearly vary with the voltage (Figure 3A) [67,68]. In Reference [67], the liquid metal marble is coated with WO_3_ and the substrate is gold. It forms an electrical interface across the n-type semiconducting coating (WO_3_) without the native oxide layer on the surface of the liquid metal, and the flow of current is owing to the combination of electron and hole carriers, which forms a *n*-type semiconductor layer. Therefore the measured current–voltage (I–V) shows the non-Ohm’s Law phenomena. They have similar I–V curves between Reference [67] and Figure 3A, however different in theory. In Figure 3A, the electronic device is composed of one gallium droplet and a layer of gold nanoparticles, and the gallium droplet is the electrode. The nanoparticle and its ligands form a capacitor. This is the process of capacitor charging and discharging. Under the threshold voltage, the circuit is not on and the current is zero. Once up the threshold voltage, the circuit is switched on. Meanwhile, some data demonstrate the relationships between the resistance, the reactance, and the frequency [57]. Figure 2A shows that when the frequency changes from 1 Hz to 10 kHz, Ga resistance remains at 0.225 Ω. However, Ga reactance closes to 0 Ω from 1 Hz to 100 Hz and has analogously exponential growth from 100 Hz to 10 kHz. Liquid metal resistance invariance under different frequencies can be applied to measure some circuit frequency changes.

### 2.2. Superior Flexibility

In order to illustrate the feasibility of liquid metal in manufacturing flexible circuits, Zheng et al. carried out a test to study the influence of bending on the stability of the circuit through measuring the resistance value when bending the printed liquid metal wire at −180°, −90°, 0°, 90°, and 180°. Figure 2C evidently indicates that the resistance variation is very little and the resistance stability of the liquid metal when bending manifests that liquid metal printed wire can be well applied for making flexible electronics [69]. Furthermore, Wang et al. bent the PVC plastic film printed by liquid metal at −180°, −90°, 0°, 90°, and 180° for one cycle, and the resistance value was measured every 50 bending cycles (Figure 2D). The resistance values fluctuated slightly from 1 to 1000 cycles, which demonstrated the stability of the liquid metal resistor line [70]. References [72,73] demonstrate that when straining the liquid metal circuits, the resonance frequency and the resistance value can be changed. All the bending and stretching tests suggest that liquid metal printed wire has reliable flexibility when employed in flexible printed electronics.

### 2.3. Tunable Adhesion

Adhesion is the attraction among different molecules, such as the adhesion between the particle and the substrate [64,65,66]. When printing a circuit, the liquid metal is ejected from the spray gun in the form of droplets [65]. When put in air, the surface of liquid metal droplets may generate a layer of oxide whose composition is Ga_2_O_3_/Ga_2_O [74]. As is disclosed on the proportional relationship between the surface tension of liquid metal droplets and oxide content [75], the smaller the metal droplets the higher the oxide content, thus the larger the surface tension and the better the substrate adhesion. Starting from this point, Zhang et al. established a generalized methodology for ubiquitous printed electronics [65] whereby, through atomized spraying of liquid metal droplets, a circuit can be quickly fabricated on almost any desired solid substrate surface, whether smooth or rough [65]. Additionally, the greater the oxide content of the metal droplets, the smaller the contact angle between the droplet and substrate [76].

Based on the Young–Dupre equation [77], one has

(8) $$W_{SL}=\gamma_{L}\left(1+\cos\theta \right),$$

where $W_{SL}$ is the adhesion work of the liquid metal drop, $\gamma_{L}$ is its surface tension, and θ is the contact angle between the droplet and substrate. The smaller the droplet, the larger the $\gamma_{L}$ and the smaller the θ, hence the greater the $W_{SL}$, representing better adhesion. In other words, one can regulate the adhesion of liquid metal ink on a substrate by controlling its oxide layer [78]. Another possible strategy would be realizing a controlled de-wetting after the metal layer has solidified by a slight temperature increase [79]. However, the liquid metal alloy ink has strong adhesion to a majority of substrates, which makes it a challenge to manufacture micro-precision circuits [80].

### 2.4. Prominent Wettability

Wettability is the ability of liquid to spread on a solid surface. The contact angle usually expresses the wettability: the smaller the contact angle the better the wettability. The dynamic contact angle is the contact angle of a liquid moving over a surface. The dynamic contact angle (sliding angle and advancing-receding angle) plays an important role in quantifying the wetting property of the oxidized Galinstan droplets compared with the static contact angle [81,82]:

(9) $$\sin\alpha =\gamma_{LG}\frac{Rk}{mg}\left[\cos\theta_{rec}-\cos\theta_{adv}\right].$$

**Figure 4:**
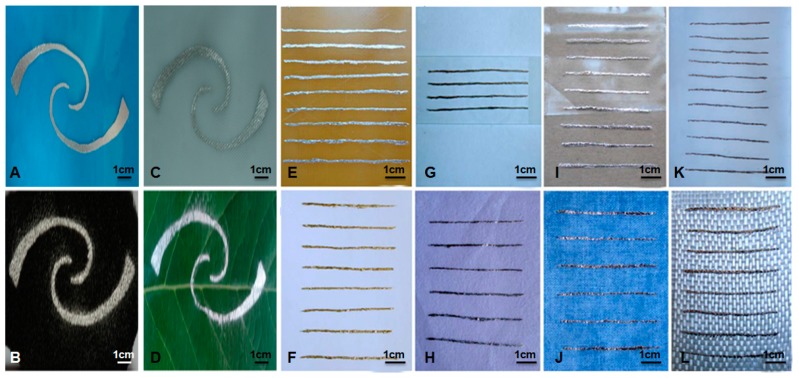
Demonstrated wettability of liquid metal printed on different substrate materials [63,75]; (**A**) Smooth polyvinylchloride; (**B**) porous rubber; (**C**) rough polyvinylchloride; (**D**) tree leaf; (**E**) epoxy resin board; (**F**) typing paper; (**G**) glass; (**H**) cotton paper; (**I**) plastic; (**J**) cotton cloth; (**K**) silica gel plate; (**L**) glass fiber cloth (Reproduced with permission from Gao, Y.; Li, H.; Liu, J., Direct writing of flexible electronics through room temperature liquid metal ink; published by PLoS ONE, 2012.) (Reproduced with permission from Zhang, Q.; Gao, Y.; Liu, J., Atomized spraying of liquid metal droplets on desired substrate surfaces as a generalized way for ubiquitous printed electronics; published by Applied Physics A: Materials Science & Processing, 2013.).

Here, α is the sliding angle; R is the liquid metal droplet radius; m is the mass of the droplet; $\theta_{rec}$ is the receding contact angle; and $\theta_{adv}$ is the advancing contact angle. The liquid metal droplet on the HCl-impregnated flattened paper has the lowest contact angle, while for the non-treated printing paper it has the highest contact angle (Figure 3B) [71]. Gao et al. have studied the excellent wettability of liquid metal GaIn_10_ with different substrate materials including smooth polyvinylchloride (Figure 4A), porous rubber (Figure 4B), rough polyvinylchloride (Figure 4C), tree leaf (Figure 4D), epoxy resin board (Figure 4E), typing paper (Figure 4F), glass (Figure 4G), cotton paper (Figure 4H), plastic (Figure 4I), cotton cloth (Figure 4J), silica gel plate (Figure 4K), glass fiber cloth (Figure 4L), and other substrates with different surface roughness and material properties [63,75]. Kramer et al. investigated the relationship between the microtextured surface topography of the liquid metal marbles and the wetting behaviors [83]. Doudrick et al. demonstrated that the oxide surface of the liquid metal alloy can improve the wettability between the metal material and substrate [80]. When increasing the oxygen content, the resistivity of the liquid metal alloy decreases while the viscosity and the wettability increase. For this reason, we can control the thickness of the oxide layer and then the wettability of the liquid metal wire on the soft substrate to obtain a superior performance of the flexible printed circuit.

## 3. Printing Technologies and Apparatuses

In ancient China, Sheng Bi, a brilliant inventor who lived around 1041 AD, invented a new type of printing method: movable-type printing, the convenience of which led to the spread of knowledge and culture; the basic principle and method of modern letterpress stamping is the same as movable-type printing in spite of its different equipment and technical conditions. Thus, the invention of an excellent printing method and the selection of appropriate printing ink can have a huge impact on human life. Table 1 shows a conductivity comparison of several typical electroconductive inks [61]. The corrosivity of the liquid metal ink for different substrates (Table 2) prevents liquid gallium from being printed on specific flexible substrates.

### 3.1. Printing Electronic Circuit

Nowadays, due to the high surface tension and good conductive ability of liquid metal, printing circuit using liquid metal ink has attracted more and more attention. The concept of DREAM ink (direct writing or printing of electronics based on alloy and metal ink) [62], as defined by Liu et al., can lead to alternative electronic devices in different fields [63,85,86]. So far, these are the different methods of printing a personal electronic circuit: screen printing [70], atomized spraying [75], microcontact printing (µCP) [87], masked deposition [88], and inkjet printing [89].

#### 3.1.1. Direct Painting or Writing

**Figure 5:**
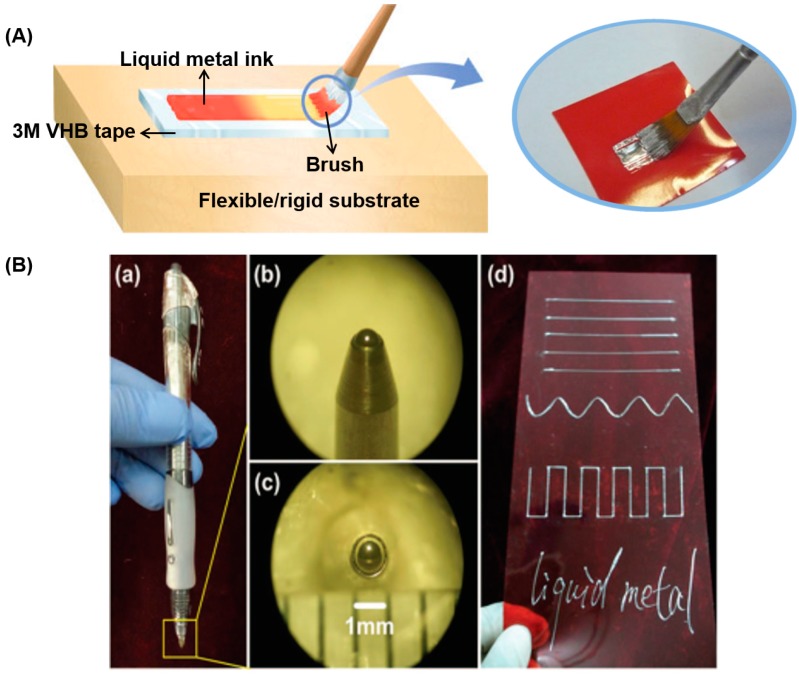
Printing methods of direct painting or writing without machine. (**A**) Direct painting of the liquid metal ink on the surface of VHB 4905 acrylic films to manufacture capacitor sensor [90]; (**B**) the liquid metal ink roller-ball pen and conductive tracks written by it [93]; (**C**) the direct writing system and the high-resolution wires written by it [73] (Reproduced with permission from Zheng, Y.; Zhang, Q.; Liu, J., Pervasive liquid metal based direct writing electronics with roller-ball pen; published by AIP Advances, 2013.).

Because of its good wettability with multifarious substrates, room-temperature liquid metal (RTLM) can be directly painted or written on paper, glass, or cloth as liquid metal ink [63]. Sheng et al. directly painted the liquid metal ink on the surface of VHB 4905 acrylic films to manufacture a capacitor sensor (Figure 5A) [90]. Because of its gravity and adhesion to substrates, the liquid metal alloy can be directly installed into the refill as a kind of ink to write conductive texts or lines [91,92]. Zheng et al. developed the liquid metal roller-ball pen (LMRP), which can write conductive lines or electronic devices on soft plates with the tip diameters ranging from 200 μm to 1000 μm (Figure 5B) [93]. Boley et al. illustrated a direct writing system, using the liquid metal alloy to fabricate small-scale stretchable electronics [73].

#### 3.1.2. Mechanical Printing Methods

**Figure 6:**
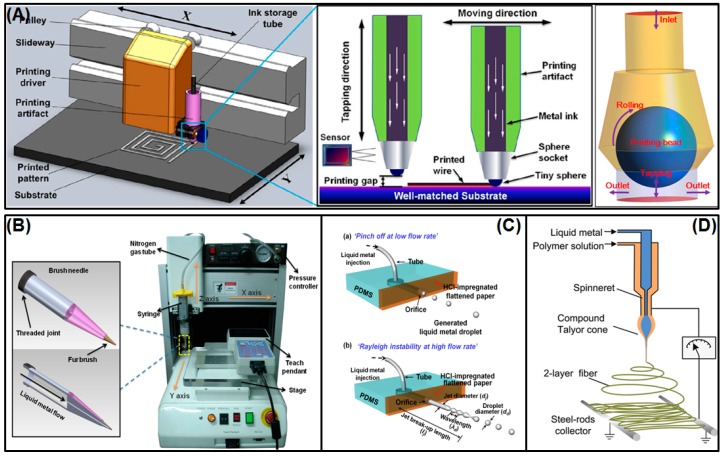
Mechanical printing methods. (**A**) The tapping mode printer and its printing process [18] (Reproduced with permission from Zheng, Y.; He, Z.-Z.; Yang, J.; Liu, J., Personal electronics printing via tapping mode composite liquid metal ink delivery and adhesion mechanism; published by Scientific Reports, 2014.); (**B**) The desktop printer to directly print circuits on paper [69] (Reproduced with permission from Zheng, Y.; He, Z.; Gao, Y.; Liu, J., Direct desktop printed-circuits-on-paper flexible electronics; published by Scientific Reports, 2013.); (**C**) inkjet printing of the liquid metal droplet based on pinch-off and Rayleigh instability [94] (Reproduced with permission from Kim, D.; Yoo, J.H.; Lee, Y.; Choi, W.; Yoo, K.; Lee, J.B.J., Gallium-Based Liquid Metal Inkjet Printing; published by IEEE Proceedings, 2014.); (**D**) coelectrospinning to fabricate a light-emitting coaxial nanofiber with a liquid metal core and polymer shell [95] (Reproduced with permission from Yang, H.; Lightner, C.R.; Dong, L., Light-emitting coaxial nanofibers; published by I ACS NANO, 2012.).

Compared to traditional direct painting or writing, mechanical printing methods can realize digital control, which makes the liquid metal flexible electronical circuits more accurate (Figure 6). Zheng et al. have manufactured and demonstrated a versatile desktop liquid metal printer that can print either a plane circuit or a 3D metal object. The machine is made up of a syringe, a nitrogen gas tube, a pressure controller, a teach pendant, a stage, the *X* axis, the *Y* axis, and the *Z* axis (Figure 6B) [69]. In order to achieve a match between different material substrate and printing ink, they have designed a brush-like porous needle to print files of different types. A desktop printer can print a variety of electrical patterns via computer software. In particular, paper is low-cost and recyclable, which makes it a common substrate material and allows the concept of printed-circuits-on-paper (PCP) [69]. Along with that, the same group created another fully automatic liquid metal printer for pervasive electronic circuit printing [18]. Its theory of tapping mode printing is fixing an ink storage tube on the printing driver, clamping the driver on the guide rail using a sliding wheel, which can make the printer head move along the guide rail in the *X* direction, and driving the base plate along in the *Y* direction using a motor (Figure 6A). Consequently, one can control the position of the printer head in the printing area of the base plate. The tapping mode means that the nib can move a small distance in the *Z* direction. When printing, the printer head falls to contact the base, uplifts a certain height over the printing substrate, so as to avoid the printed line. In the process of printing, the printing header bead rotates due to the basal friction, which makes the liquid metal ink in the pen container run downwards. One can use the tapping mode method to manufacture various printed-circuit-on-board (PCB) circuits, antennae, and so on. In addition, other mechanical system printing methods to print personal electronic circuits using liquid metal include inkjet printing and coelectrospinning (Figure 6C,D) [89,94,95].

#### 3.1.3. Mask-Based Printing Methods

**Figure 7:**
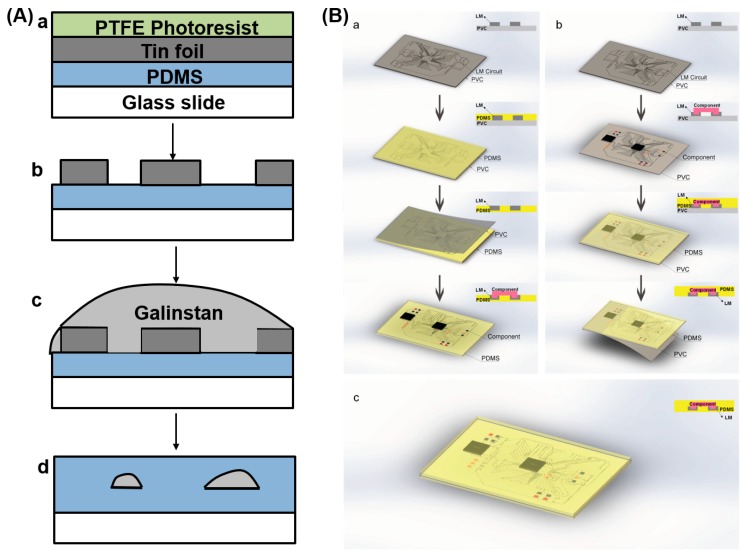
Mask-based printing methods. (**A**) The masked deposition process of gallium-indium alloys for liquid-embedded elastomer conductors, (**a**) painting photoresist onto tin foil; (**b**) micro-channel on the elastomer surface; (**c**) flooding the surface with galinstan; (**d**) removing excessgalinstan with a thin film, cooling the liquid metal in a freezer and encapsulating it by coating (Modified from [88]); (**B**) the process of dual-trans printing fabrication [58] (Reproduced with permission from Wang, Q.; Yu, Y.; Yang, J.; Liu, J., Fast fabrication of flexible functional circuits based on liquid metal dual-trans printing; published by Advanced Material, 2015.).

**Figure 8:**
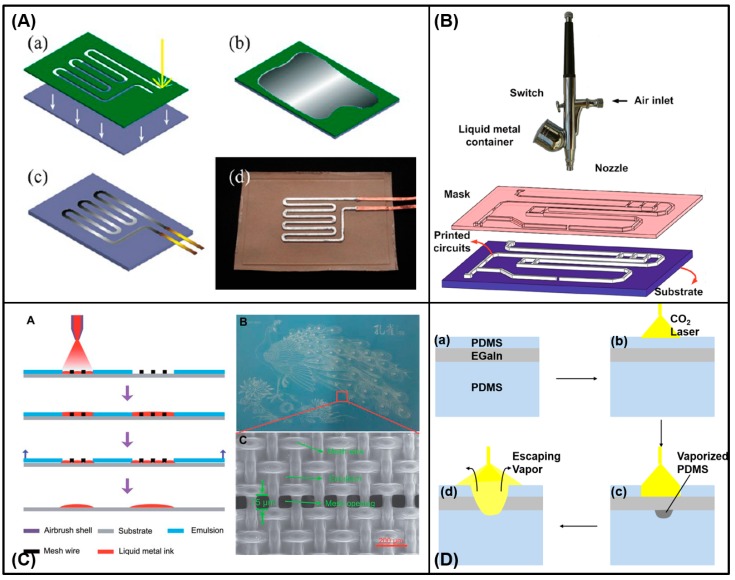
Mask-based printing methods. (**A**) The microcontact printing process to fabricate the liquid metal soft-matter circuit [87], (**a**) laser engraving a thin film; (**b**) spreading GaIn alloy over the stencil; (**c**) removing the stencil and inserting the copper wires before sealing; (**d**) unsealed galinstan heater on VHB elastomer produced with stencil lithography (Reproduced with permission from Tabatabai, A.; Fassler, A.; Usiak, C.; Majidi, C., Liquid-phase gallium–indium alloy electronics with microcontact printing; published by Langmuir, 2013.); (**B**) atomized spraying of liquid metal droplets to rapid prototype circuits [75] (Reproduced with permission from Zhang, Q.; Gao, Y.; Liu, J., Atomized spraying of liquid metal droplets on desired substrate surfaces as a generalized way for ubiquitous printed electronics; published by Applied Physics A: Materials Science & Processing, 2013.); (**C**) the screen printing process and scanning electron microscopy image of the screen mesh [70] (Reproduced with permission from Wang, L.; Liu, J., Pressured liquid metal screen printing for rapid manufacture of high resolution electronic patterns; published by RSC Advances, 2015.); (**D**) proposed CO_2_ laser ablation mechanism of the liquid metal alloy on the PDMS substrate (Modified from [96]).

Printing methods based on masks are the most commonly used method to manufacture liquid metal flexible electronics in the laboratory. Kramer et al. provided a masked deposition method to fabricate liquid metal hyperelastic electronic circuits [88] (Figure 7A). At the same time, Gozen et al. used the masked deposition method to prepare micro-scale high-density soft-matter electronics [66]. Wang et al. proposed a rapid fabrication of flexible functional circuits based on liquid metal dual-trans printing, which can be applied to quickly fabricate a flexible functional electronic device fitting to any complex surface shape (Figure 7B) [58]. First one can print out a liquid metal circuit on PVC membrane, then use polydimethylsiloxane (PDMS) solution to cover the circuit. After a period of time, PDMS solution can be cured and when it is liquid, arbitrary shape objects can be immersed into PDMS solution. Finally, cool the whole object to make liquid metal solid and one can completely transfer the initial liquid metal circuit to a PDMS flexible substrate. In the process, after PDMS curing, one can peel off the PVC membrane and target object to obtain PDMS device embedded liquid metal flexible circuits. Because the shape of the PDMS substrate completely fits with objects, one can achieve highly conformal electronic devices. This technology has significant implications for sensing, monitoring in health care, and households as a corresponding device to conduct specific functions. Common methods mentioned in the literature also include microcontact printing [87], atomized spraying [75], screen printing [70], and CO_2_ laser ablation [96] (Figure 8D). The theory of the atomized spraying and the screen printing methods is that the gun sprays the liquid metal ink onto the substrate through a specific mask to form a circuit which has the same shape as the mask (Figure 8B,C).

**Figure 9:**
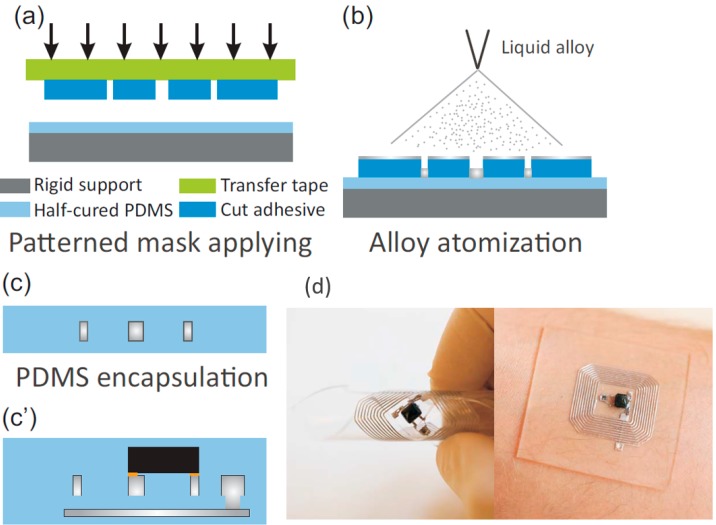
Processing steps of manufacturing liquid alloy microfluidic wireless power transfer (**a**–**c**), an active component mounted in the circuit followed by encapsulation (**c’**) and in rolled state and attached to human arm (**d**) [97] (Reproduced with permission from Jeong, S.H.; Hjort, K.; Wu, Z., Tape transfer atomization patterning of liquid alloys for microfluidic stretchable wireless power transfer; published by Scientific Reports, 2015.).

The common method for preparation of liquid metal microfluidic electronics is pouring the liquid metal alloy into a specific shape of channel to form the functional circuit. Therefore, from the strict definition, the preparation method of microfluidic electronics cannot be classified as fabrication of liquid metal flexible printed electronics. However, Figure 9 demonstrates the processing steps of manufacturing liquid alloy microfluidic wireless power transfer, in which the mask layer and atomization printing technology are applied and the power transfer displays large flexibility in its rolled state and can attach to the human arm [97].

#### 3.1.4. High-Resolution Nanoimprint Lithography

Unlike the relatively large liquid metal droplets, one can also prepare liquid metal nanoparticles to print micro-scale or nano-scale wires. John et al. produced small features through mechanically sintering gallium–indium nanoparticles to realize high-resolution nanoimprint lithography [98]. As shown in Reference [98], 1 μm coalesced EGaIn line arrays were formed by reducing the size of the sintering tool. Via inkjet printing EGaIn nanoparticles dispersions on the surface of nitrile glove, the functionalized elastomer glove with liquid metal micro-wire arrays represents excellent stretchability when holding a tennis ball.

### 3.2. 3D Printing

**Figure 10:**
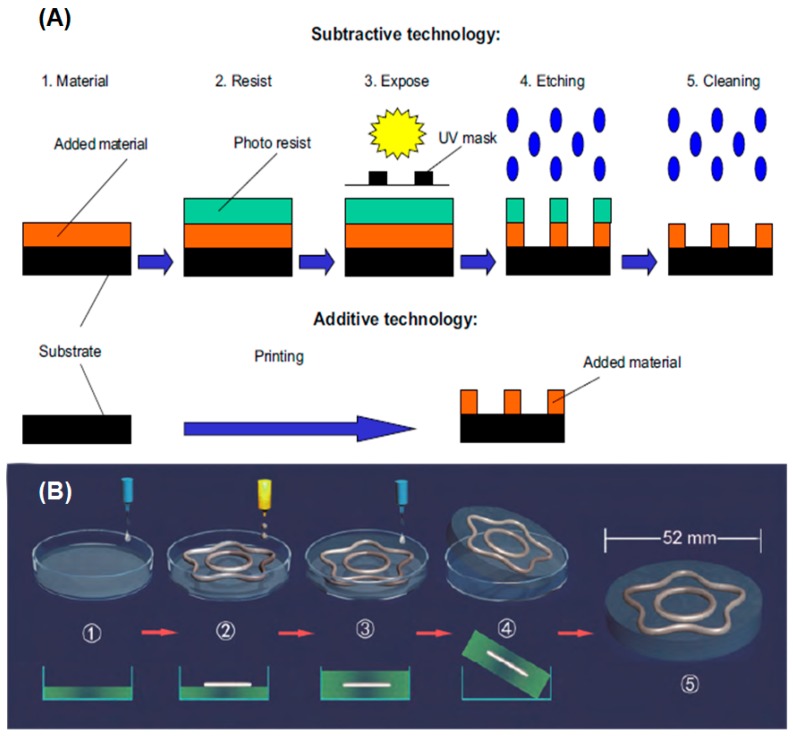
(**A**) Difference between subtractive and additive manufacturing technologies [99] (Reproduced with permission from Kunnari, E.; Valkama, J.; Keskinen, M.; Mansikkamäki, P., Environmental evaluation of new technology: Printed electronics case study.; published by Journal of Cleaner Production, 2009.); (**B**) hybrid 3D printing process with Bi_35_In_48.6_Sn_16_Zn_0.4_ and 705 silicone rubber inks [64] (Reproduced with permission from Wang, L.; Liu, J., Compatible hybrid 3D printing of metal and nonmetal inks for direct manufacture of end functional devices; published by Science China Technological Sciences, 2014.).

3D printing is a technology to construct an object by means of printing layer by layer; in other words, it is a kind of typical additive manufacturing technology. Different from traditional subtractive technology, additive manufacturing is a top-down, layer-by-layer manufacturing process. It can not only avoid corrosion and contribute to environmental protection, but also saves a lot of raw materials. This significantly reduces environmental pollution and the cost of materials. Figure 10A shows the difference between subtractive and additive manufacturing technologies [99]. Next we will introduce several 3D printing technologies using low melting point metal ink based on the additive mode.

**Figure 11:**
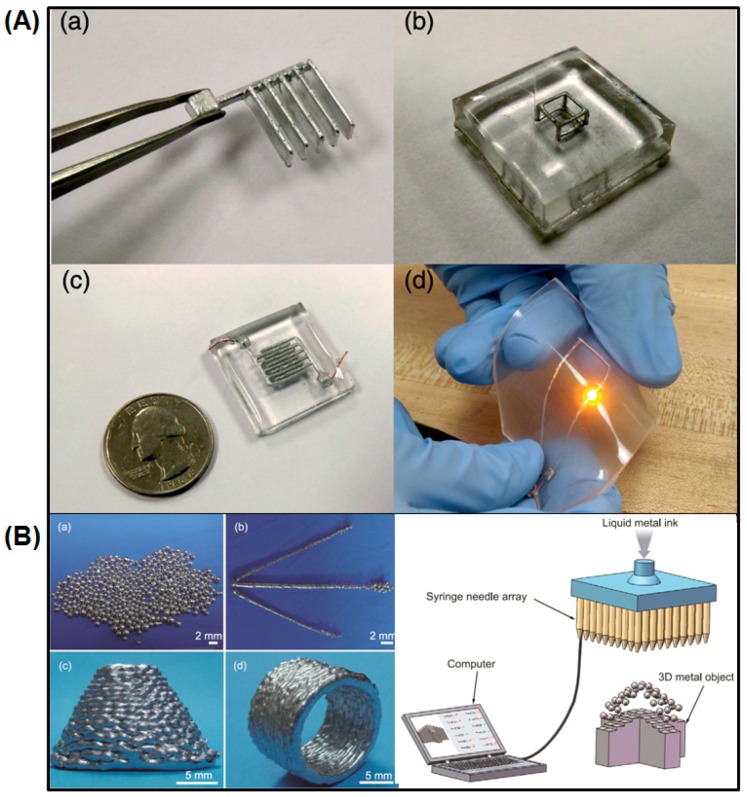
(**A**) The electronic circuits and devices created by freeze casting [101] (Reproduced with permission from Fassler, A.; Majidi, C., 3D structures of liquid-phase gain alloy embedded in pdms with freeze casting; published by Lab on a Chip, 2013.); (**B**) liquid phase 3D printing method [100], (**a**) liquid metal balls; (**b**) liquid metal rods; (**c**) cone structure; (**d**) cylinder structure (Reproduced with permission from Wang, L.; Liu, J., Liquid phase 3D printing for quickly manufacturing conductive metal objects with low melting point alloy ink; published by Science China Technological Sciences, 2014.).

Wang et al. first put forward the concept of liquid phase 3D printing to employ gallium-, bismuth-, and indium-based alloys whose melting points are higher than room temperature and lower than 300 °C to accomplish the printing process [100]. The liquid phase 3D printing is achieved in a liquid environment, and the liquid can be water, ethanol, kerosene, electrolyte solution, etc. However, the melting point of liquid metal ink must be lower than that of the liquid environment to ensure the printed item run. In the experiments, Wang et al. introduced Bi_35_In_48.6_Sn_16_Zn_0.4_ as printing ink because it does not absorb/release as much heat as normal metal in the process of phase change (Figure 11B) [100]. Shortly after, they originated a late-model 3D printing technology, hybrid 3D printing, which means interactive printing of various inks or combination of multiple printing methods. In Figure 10B, Bi_35_In_48.6_Sn_16_Zn_0.4_ and 705 silicone rubber (nonmetal) inks were adopted to accomplish hybrid 3D printing [64]. The 705 silicone rubber serves as a kind of waterproof, anticorrosive, transparent, and insulating adhesive, and can be solidified when absorbing water vapor at room temperature. Therefore, it is often used as an electrical packaging material. First, one can print the bottom layer on the substrate using 705 silicone rubber and print the middle layer on the 705 silicone rubber layer using Bi_35_In_48.6_Sn_16_Zn_0.4_. After that, one can print the top layer using 705 silicone rubber. When adequately solidified, a sandwich structure can be obtained once taking off the printed item from the basement. Compatible hybrid 3D printing of metal and nonmetal inks fully embodies the good mechanical strength and electrical/thermal conductive of metal ink, and excellent insulation of nonmetal ink, which makes the printing circuit applicable in a harsh environment. Fassler et al. applied the common freeze-casting method to manufacture various liquid metal based 3D structures (Figure 11A) [101]. Ladd et al. tested the direct-writing microcomponents of the liquid metal alloy for 3D free-standing liquid metal microstructures at room temperature [102]. Liquid metal can also be employed as an injectable 3D bio-electrode to measure the electrocardiograph (ECG) or electroencephalogram (EEG) [54,57,103].

### 3.3. Printing Devices

Liu et al. have delivered printers that have now been put into practical use (Table 3) [18,100]. Liquid metal circuit printers have completely changed the traditional mode, breaking the technical bottleneck on personal electronics manufacturing, which makes it a reality to quickly fabricate electronic circuits with extremely low costs. Through computer control, even people with no electronics experience can download a circuit diagram from the Internet and print it directly. Inkjet printing equipment is based on the liquid metal screen printing process. Through a USB cable, the printer can connect with a computer to realize online printing, and can also print offline via an SD card. Before printing one needs to prepare .gcode files to control the nozzle movement. A 3D Metal Printer is a desktop fused deposition modeling (FDM) device; it substitutes traditional metal parts processing to prepare metal parts with a complex spatial structure.

## 4. Typical Applications

As a novel electronic ink material, the flexibility and shape-preserving of the liquid metal alloy allows it to play an important role in fabricating components and soft circuit. Its non-toxicity and benign biocompatibility make it an implant material in the body, with uses such as the selected low melting point metal skeleton material [51], high-resolution angiography contrast [52], biological micro-droplets [95], nerve connection [35,36], human exoskeleton [104], and electronic skin [55,56,105].

The technology of directly printing liquid metal electronic circuit transforms conventional rigid circuits process to flexible desktop manufacturing [106]. With a way to rapidly manufacture various flexible household electronics with different functions, one can envisage that daily life will undergo big changes in the near future. Imagine a quilt with flexible functional circuits containing temperature or pressure sensors that can monitor human sleep quality and adjust the quilt temperature according to body temperature to achieve intelligent heating or cooling. In fact, all of these needs can be implemented by liquid metal flexible circuits. Liquid metal flexible printed electronics can be directly printed on many soft substrates, and could be used to produce lots of complex artwork. Zhang et al. demonstrated that even leaves can become a circuit board, which provides a solid theoretical basis for the universality of liquid metal atomized spraying manufacturing flexible electronics [75].

Through the direct-writing method, Li et al. drew a liquid metal thermocouple on paper [85]. The liquid metal thermocouple has many special advantages, such as small contact resistance and thermal resistance, no need for welding, and a wide working temperature zone. The thermoelectric effect is the theoretical basis of thermocouple temperature measurement. The thermoelectric power produced by the thermocouple has nothing to do with its shape or size, but relates to the composition of the thermoelectric materials and the temperature difference between the two ends. The thermocouple composed of Ga and EGaIn not only has temperature sensing ability but also bending capacity.

Fassler et al. prepared two kinds of capacitors—the square wave capacitor and the spiral capacitor—and a square planar spiral inductor [107]. They calculated the values of the capacitors or inductor of various shapes via formula derivation. A soft-matter diode, a new type of flexible functional equipment component with liquid metal electrodes, was developed by So et al. [29]. Because the liquid metal EGaIn electrodes’ sandwich layers are conductive forward from Ga*_x_*O*_y_* to Ga while nonconductive backward from Ga to Ga*_x_*O*_y_*, the soft-matter diode has the function of limiting current and can pass the current one-way through.

**Figure 12:**
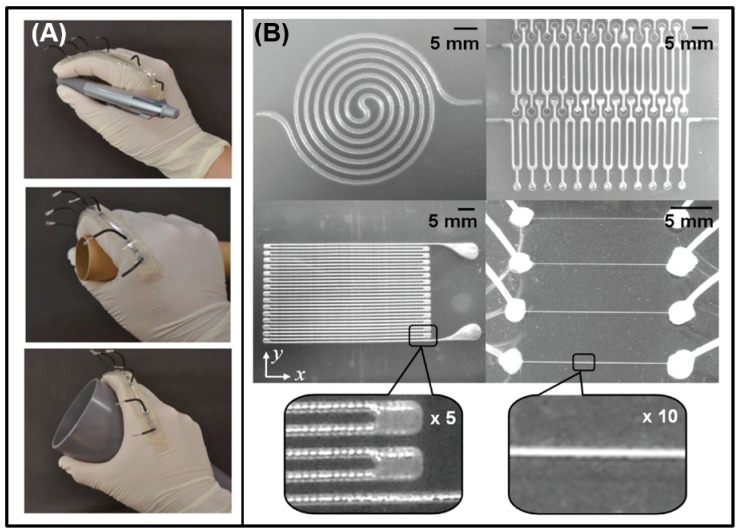
Sensors based on liquid metal deformation. (**A**) A wearable data glove with local strain sensing using GaInSn elestrodes [59] (Reproduced with permission from Matsuzaki, R.; Tabayashi, K., Highly stretchable, global, and distributed local strain sensing line using gainsn electrodes for wearable electronics; published by Advanced Functional Material, 2015.); (**B**) hyperelastic pressure sensors with microchannels of conductive liquid metal alloy [114] (Reproduced with permission from Park, Y.-L.; Majidi, C.; Kramer, R.; Berard, P.; Wood, R.J., Hyperelastic pressure sensing with a liquid-embedded elastomer; published by Journal of Micromechanics and Microengineering, 2010.).

**Figure 13:**
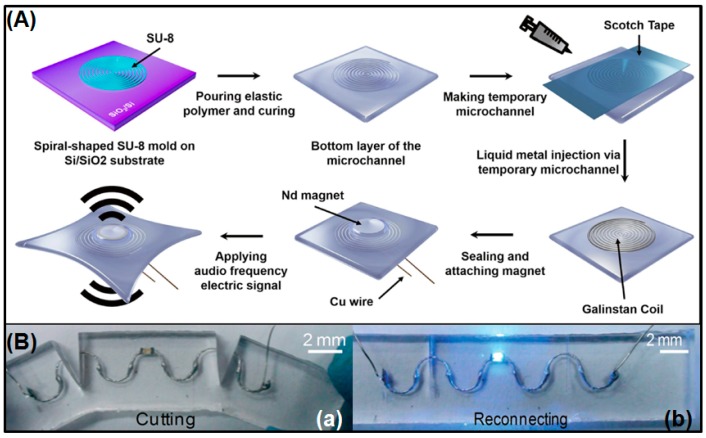
The liquid metal flexible printed electronics functional devices. (**A**) The fabrication process of the liquid metal stretchable loudspeaker [112] (Reproduced with permission from Jin, S.W.; Park, J.; Hong, S.Y.; Park, H.; Jeong, Y.R.; Park, J.; Lee, S.-S. ; Ha, J.S., Stretchable loudspeaker using liquid metal microchannel; published by Scientific Reports, 2015.); (**B**) Physical separation (**a**) and reconnection (**b**) of the liquid metal circuit with an LED [113] (Reproduced with permission from Li, G.; Wu, X.; Lee, D.-W., A galinstan-based inkjet printing system for highly stretchable electronics with self-healing capability; published by Lab on a Chip, 2016.).

At the same time, because of its flexibility and fluidity, liquid alloy is widely used in sensors and functional devices; Wu’s group made important contributions to the field [108]. They revealed a liquid alloy microfluidic stretchable large-area wireless strain antenna, consisting of two layers of liquid metal alloy filled microfluidic channels in a silicone elastomer [109]. A stretchable ultra-high frequency radio frequency identity tag printed on human skin demonstrated that liquid alloy microfluidic electronics can be applied to electronic skin [56]. Liquid alloy microfluidic electronics can also be fabricated as antennae with strong stretchability, rolled space [110,111], and liquid alloy microfluidic wireless power transfer [97]. Matsuzaki et al. designed a wearable data glove with local strain sensing using GaInSn elestrodes (Figure 12A) [59]; the data glove can judge the degree of finger bending according to the change in resistance at different monitoring points. Krupenkin et al. modeled a footwear-embedded microfluidic energy harvester, which is a wearable mechanical-to-electrical energy conversion based on liquid metal microdroplets [60]. Meanwhile, the liquid metal Galinstan can fabricate a stretchable loudspeaker to apply audio frequency electric signal (Figure 13A) [112]. When the liquid metal circuit is cut off, once reconnected, it can work well without additional repairmen (Figure 13B) [113].

## 5. Discussion and Conclusions

As new-generation inks for flexible printed electronics, liquid metals possess outstanding versatility and specific merits of fluidity, conductivity, metallicity, and electromagnetic properties. They thus offer tremendous opportunities for making future flectional electronic devices. However, there are still challenges in preparing, processing, and utilizing high-performance inks. For example, the low adhesion to many substrates, easy oxidation, and high surface tension of liquid alloy inks still restrict the realization of high-precision, stable circuits. To solve such key issues, we can mix the liquid metal with materials that are metal or non-metal. Therefore, new hybrid functional liquid metal materials can be made with desired properties of semiconductivity, semi-flexibility, enhanced adhesion, antioxidant ability, etc. This would enable the inks to adapt well to practical situations and have more functions. For example, the liquid metal nanocrystallization can improve printing precision, and one can modify the surface of liquid metal nanoparticles, e.g., by combining with the luminous substance through metal bond to achieve the self-luminous colorful printing.

So far, liquid metal ink has already been printed on a wide range of soft substrates, such as plate, paper, cloth, skin, leaves, and so on, which opens up many opportunities for making flexible electronical components like sensors, actuators, wearable bioelectronics, electrical skin, etc. The use of such diverse flexible substrates significantly enriches the application field of liquid metal flexible printed electronics, and one can thus expand related practices from human beings to other organisms such as animals and plants. For example, one can even print flexible circuits with features of energy harvesting and light emission on leaves to capture the energy of swinging leaves to supply light at night.

Various kinds of liquid metal printing technologies are enabling efficient circuit fabrication. At this stage, a series of technological problems still need to be tackled, such as precise and reliable connection between the printed liquid metal circuits with the traditional integrated circuits. High precision printing is critical to achieve the liquid metal integrated circuits. Regarding the specific fabrication, hybrid flexible printing with planar printing and 3D printing are worth trying. Furthermore, cooling of complex liquid metal circuits should be carefully monitored to avoid damage from the “thermal barrier.” Meanwhile, encapsulation is often a necessity for making ending user devices. For instance, long-lasting liquid metal skin electronics mainly depend on packaging technology. PDMS is often applied to package liquid metal flexible circuits. However, for various substrates in different environments, one needs to consider diverse encapsulation technologies to make the circuit more secure in order to achieve the desired functionality. For applications in organisms, the non-toxicity of packaging materials especially needs to be considered. Sometimes, liquid metal’s movement and shape-shifting under the electric field can produce more self-configurational operations of the circuits [106,107]. These unconventional, dynamically changing or self-destructive circuits are also worth pursuing.

In summary, liquid metal flexible printed electronics break through the limitations of traditional flexible electronics. The process of circuits manufacturing is straightforward, fast, stable, and relatively compatible with existing integrated circuit technology. Liquid metal flexible printed electronics would allow us to make high-quality products such as wearable devices, electronic skin, medical implants, flexible display, and solar panels that will meet the coming demands. The existing liquid metal flexible printed technology is a combination of a liquid metal soft circuit and rigid components or chips, so not completely flexible electronics in the larger sense. At the same time, this kind of semi-flexible circuit may partially limit its application. A basic significance of liquid metal flexible printed electronics lies in the popularity of liquid metal printing technology: people can discretionarily print their personalized flexible functional devices on any surface, which can significantly expand traditional electronics engineering. By overcoming the problems in obtaining a high rate of good product, self-repair of damaged circuits, long service life, low environmental pollution, and waste recycling, liquid metal flexible printed electronics can contribute to a bright future.

## Figures and Tables

**Table 1:** A comparison of the electrical conductivities of several typical conductive inks [61].

Ink Type	Ink Composition	Conductivity
Carbon conductive ink	Carbon	1.8 × 10^3^ S/m
	CNT	(5.03 ± 0.05) × 10^3^ S/m
Polymer conductive ink	PEDOT:PSS	8.25 × 10^3^ S/m
Nano-silver ink	Ag-DDA	3.45 × 10^7^ S/m
	Ag-PVP	6.25 × 10^6^ S/m
Liquid metal ink	EGaIn	3.4 × 10^6^ S/m
	Bi_35_In_48.6_Sn_16_Zn_0.4_	7.3 × 10^6^ S/m

**Table 2:** The corrosion between liquid gallium and other metals [84].

Corrosive Behavior	Easy to React with Gallium	Better Resistance for Corrosion	Best Resistance for Corrosion
Metal	Iron, nickel, cadmium, cerium, copper, aluminum, gold, manganese, platinum, silver, tin and vanadium, zirconium	Titanium, tantalum, niobium, molybdenum, beryllium	Tungsten, rhenium, sintering BeO, Al_2_O_3_, and fused quartz, graphite
Temperature range	Appearing corrosion at 200 °C or even lower	Appearing corrosion above 400 °C	No corrosion even at 800 °C

**Table 3:** Three kinds of printers [69] (Reproduced with permission from Zheng, Y.; He, Z.; Gao, Y.; Liu, J., Direct desktop printed-circuits-on-paper flexible electronics; published by Scientific Reports, 2013.).

Liquid Metal Printer	Personal Circuit Printer	Ubiquitous Inkjet Printer	3D Metal Printer
Device type	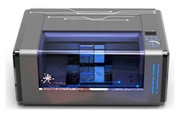	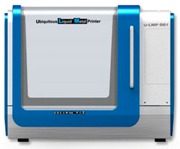	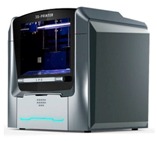
Printed out items	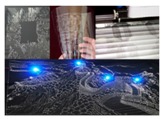	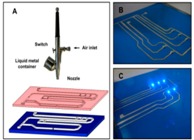	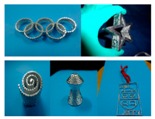
Application Fields	circuit; art; devices; rapid prototyping.	electronic circuit; art; radio frequency identification label; antenna.	circuit; radar; antenna; molds.
